# Fibrinolytic Administration via EkoSonic™ Endovascular System (EKOS) Catheter Used in Acute Aortic Occlusion Secondary to COVID-19

**DOI:** 10.7759/cureus.16893

**Published:** 2021-08-04

**Authors:** Harrison Moynihan, Jordan Richardson, Kristian Loveridge

**Affiliations:** 1 Internal Medicine, Detroit Medical Center, Detroit, USA; 2 Emergency Medicine, Detroit Medical Center, Detroit, USA; 3 Interventional Radiology, Detroit Medical Center, Detroit, USA

**Keywords:** covid-19, acute aortic occlusion, fibrinolytics, ekos catheter, thrombosis

## Abstract

Severe acute respiratory syndrome coronavirus 2 (SARS-CoV-2) or COVID-19 is the virus responsible for the 2019 global pandemic. Pulmonary complications of COVID-19 are well established in the literature. However, the virus causes numerous extrapulmonary manifestations, notably acute aortic occlusion (AAO). COVID-19 creates a hypercoagulable state via the upregulation of numerous procoagulant cytokines in endothelial cells of blood vessels. We present a case of a 63-year-old patient without a previous history of prothrombotic disorders who developed AAO in the distal abdominal aorta and bilateral common iliac arteries after contracting COVID-19. The patient was a poor surgical candidate and was treated with fibrinolytics that were administered via an EkoSonic™ Endovascular System (EKOS) catheter using a bilateral transfemoral approach. This case highlights a unique treatment option for non-surgical candidates with AAO.

## Introduction

Acute aortic occlusion (AAO) has a multitude of etiologies including aortic emboli, thrombosis of an atherosclerotic plaque, aortic stent occlusion and aortic dissection [1,2]. Acute aortic occlusion has also been linked as an extrapulmonary manifestation of severe acute respiratory syndrome coronavirus 2 (SARS-CoV-2) or COVID-19. The virus creates a hypercoagulable state by upregulating procoagulant cytokines within endothelial cells of blood vessels [3]. 

Acute aortic occlusion can be managed surgically or non-surgically depending on the health status of the patient. Bilateral transfemoral embolectomy, aortobifemoral bypass and thrombolysis are all viable treatment options that are well described in the literature [1,2].

We present a case of a 63-year-old patient that developed AAO secondary to COVID-19 infection. This case represents a unique way of treating AAO using fibrinolytics administered through an EKOS catheter using a bilateral transradial approach. 

## Case presentation

A 63-year-old female with newly diagnosed atrial fibrillation with rapid ventricular response (RVR), history of type 2 diabetes, hypertension, and deep venous thrombosis six years ago presented to the emergency department with paresthesia of the left lower extremity. She had been feeling sick over the last few days with a sore throat, cough, and shortness of breath. She denied any chest pain.

Physical examination revealed absence of pulses in the left lower extremity and coolness to the touch. Initial laboratory workup in the emergency department was performed, and significant findings are illustrated in table 1. Initial differential diagnosis is broad and includes acute COVID-19 pneumonia, acute diabetic ketoacidosis, acute atrial fibrillation with RVR, and acute limb ischemia.

**Table 1:** Significant initial laboratory findings in emergency department workup

Lab Marker	
Troponin	49 ng/mL
D-dimer	23.02
Carbon dioxide	11 mEq/L
Creatinine	1.98 mg/dL
Glucose	657 mg/dL
Lactic Acid	9.1 mmol/L
White Blood Cell (WBC) Count	16.9 x 10^9^/L
Rapid SARS (COVID-19) PCR	Positive

**Figure 1:**
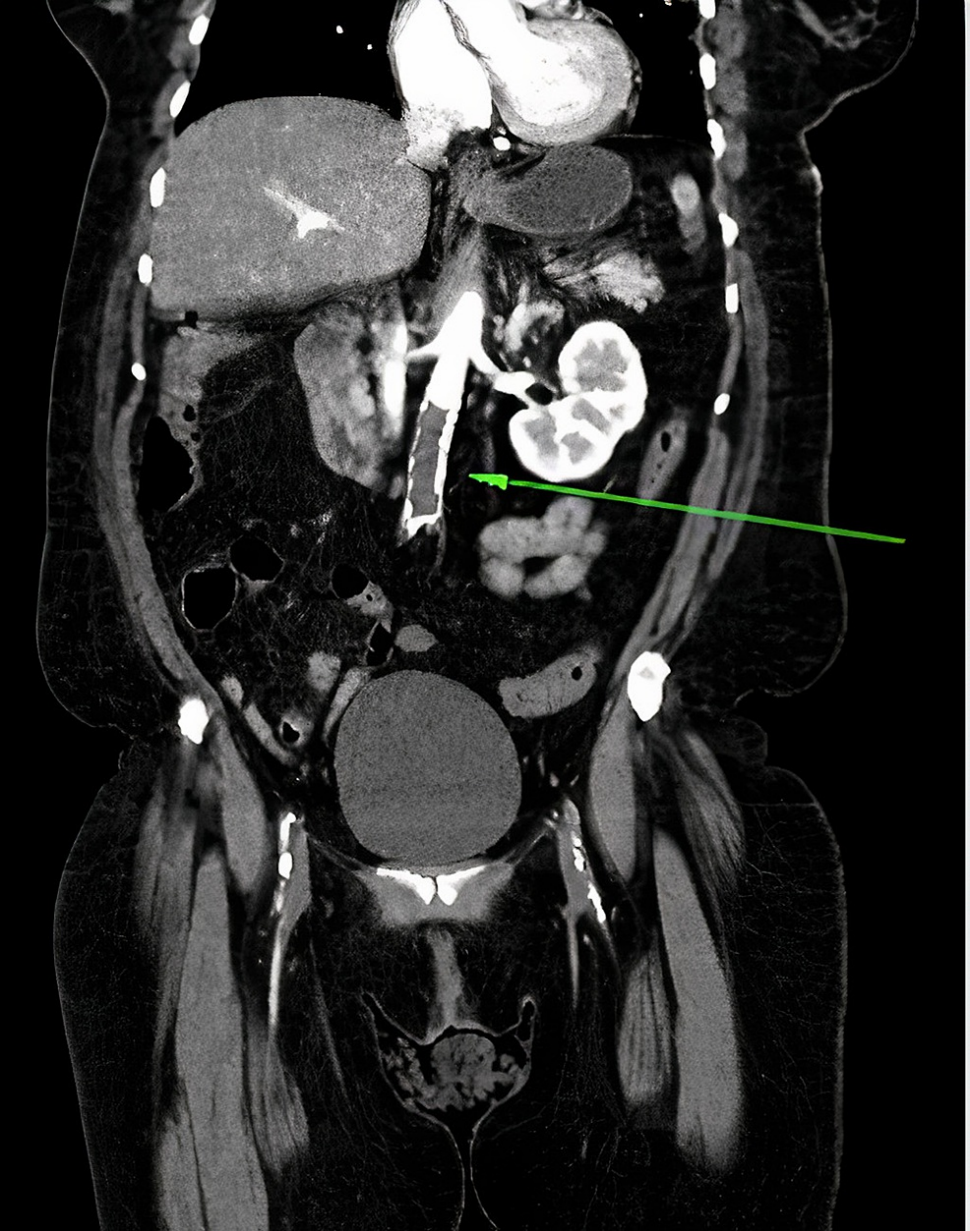
CT abdomen and pelvis with contrast demonstrating a large filling defect inferior to the renal arteries which extends to the level of the proximal superficial femoral artery bilaterally (Green Arrow). Image courtesy of Kristian O. Loveridge, D.O.

**Figure 2:**
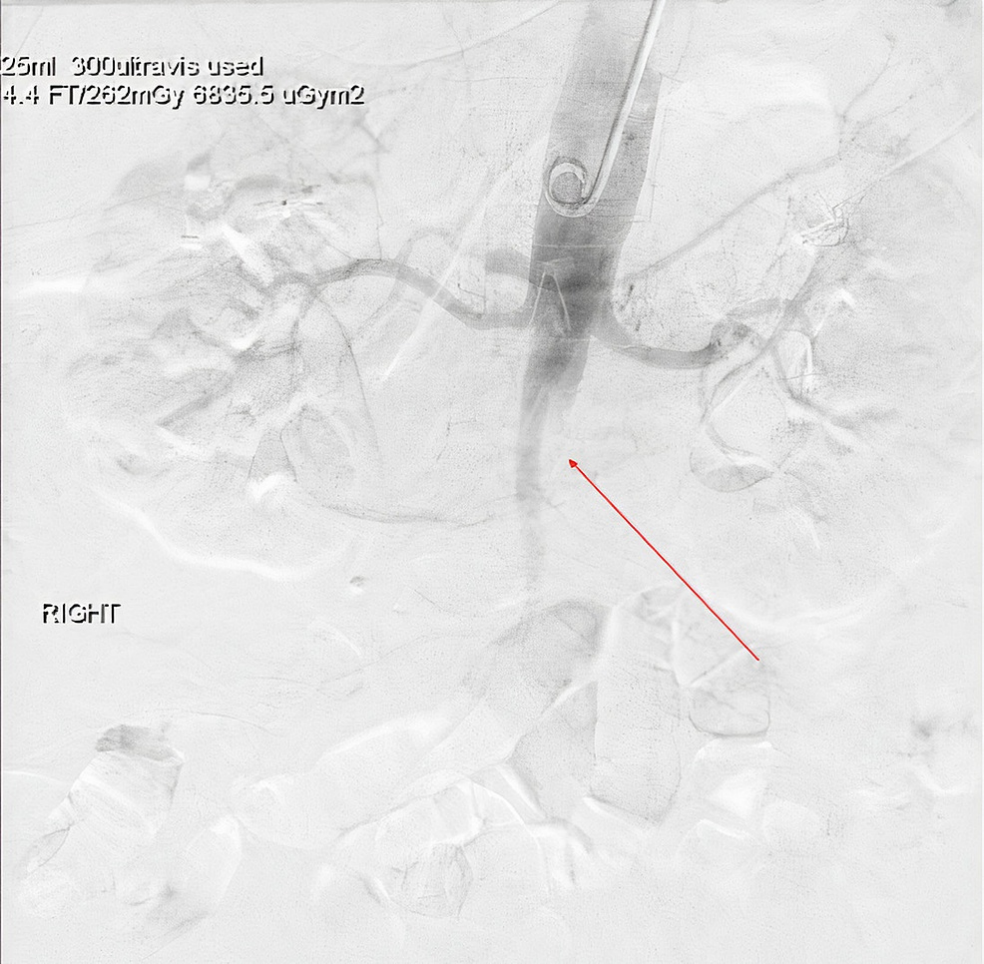
Fluoroscopy with contrast demonstrating patent bilateral renal arteries and distal infrarenal aorto-occlusive disease with major filling defect (Red arrow). Image courtesy of Kristian O. Loveridge, D.O.

A chest radiograph demonstrated asymmetric haziness in the right lower lateral lung field. A Computed Tomography (CT) scan of the thorax was obtained and showed bilateral pulmonary ground glass opacities and basilar atelectasis, but demonstrated no evidence of pulmonary embolism. Next, a CT of the abdomen was obtained and showed a 1.7 x 1.0 cm filling defect superior to the celiac artery in the abdominal aorta as well as a large occlusive filling thrombus defect just inferior to the renal artery to the level of the common femoral artery bilaterally (Figure 1). The aortic thrombus was also appreciated under fluoroscopy as illustrated in (Figure 2).

In this case, the patient was deemed a poor surgical candidate due to disease severity and comorbid conditions. Therefore, a treatment approach via thrombolysis was elected over traditional surgical bypass or thrombectomy.

**Figure 3:**
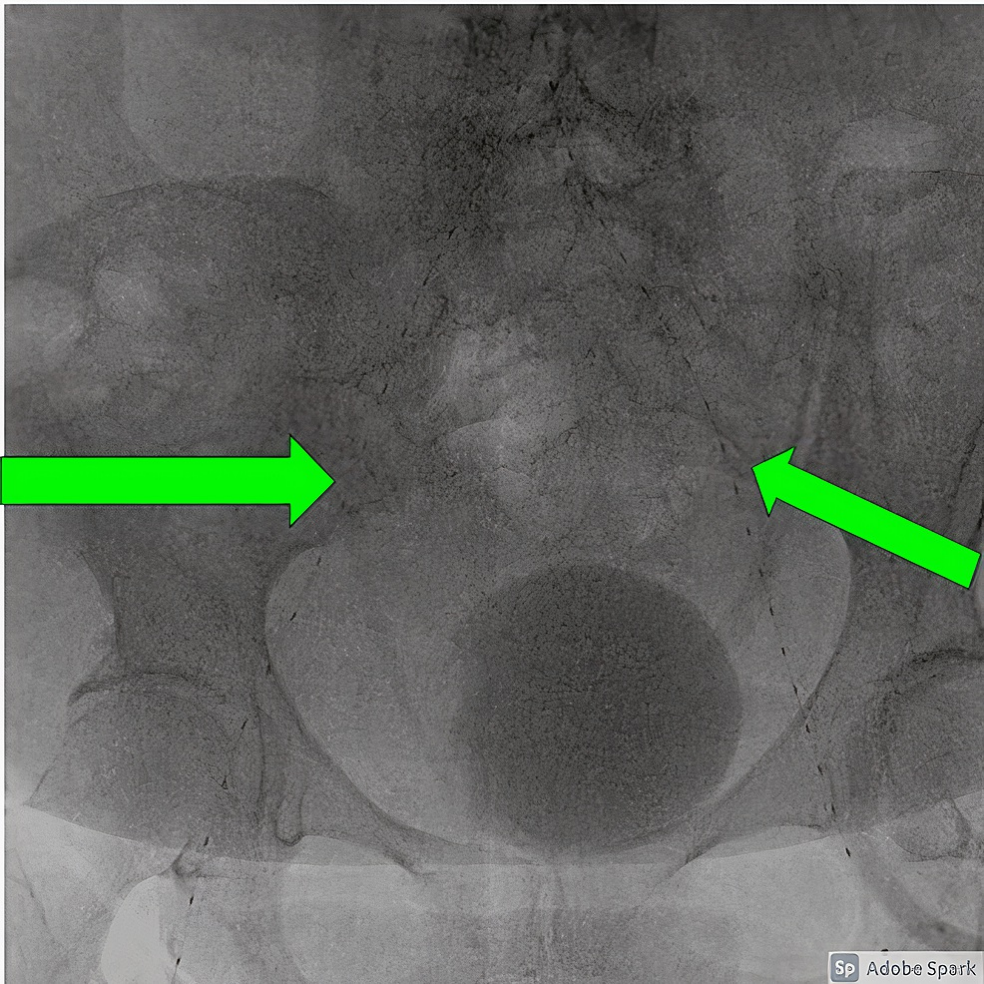
EKOS catheters placed across the aortic thrombus with the infusion segment of the EKOS catheters extending into the bilateral superficial femoral arteries (Green Arrows). Image courtesy of Kristian O. Loveridge, D.O.

Bilateral percutaneous radial artery access was obtained because the common femoral arteries were nearly completely occluded with thrombus. This approach was unique because access is traditionally gained via the femoral arteries. Angiography of the abdominal aorta demonstrated patent suprarenal segment with good perfusion to the patient's bilateral renal arteries. However, complete thrombotic occlusion below the level of the renal arteries was present. No flow was visualized into the pelvic arteries. Selective angiography of the bilateral external iliac arteries demonstrated stasis of flow. Bilateral transradial EKOS thrombolysis catheters were placed within the aorta, extending into the lower extremities (Figure 3). The infusion side-holes were positioned to span the aortoiliac thrombus. Heparin and tissue plasminogen activator were initiated for thrombolysis.

The patient expired hours later from cardiopulmonary arrest, likely from severe COVID-19 infection and multiorgan dysfunction but also exacerbated by reperfusion injury. It is unknown whether or not the patient was vaccinated against COVID-19.

## Discussion

COVID-19 patients are at increased risk of vascular complications such as arterial and venous thrombi as well as cardiac dysfunctions including arrhythmias and myocarditis [4,5]. COVID-19 can directly infect endothelial cells causing inflammation and apoptosis, resulting in the formation and release of pro-coagulant cytokines and acute phase reactants including fibrinogen and thrombin [3].

While in-situ aortic thromboses are exceedingly rare, there have been increased reports of COVID-19 resulting in thrombi in abnormal locations such as the aorta [3]. The patient described in this case developed an AAO secondary to severe COVID-19 infection, which then resulted in thrombotic occlusion of the bilateral common iliac arteries due to thrombus propagation.

Acute aortic occlusion can be treated medically, surgically, or percutaneously. Often, a combination of these techniques is employed. For patients diagnosed with AAO, heparin therapy is initiated as soon as possible [6]. The established gold standard procedure for treating AAO is the bilateral transfemoral thrombo-embolectomy [7,8]. This is a favorable approach, especially for patients who are poor surgical candidates since laparotomy is avoided. In 2019, Grip et al. studied treatment modalities and outcomes in patients with AAO and determined that thrombo-embolectomy was the most frequently employed method of revascularization [9]. If this treatment modality is unsuccessful, aortobiiliac/bifemoral bypass is the next best treatment option provided the patient is stable enough to undergo the operation [7]. For high risk patients, extra-anatomical bypass such as axillobifemoral bypass is the treatment of choice due to the increased morbidity and mortality associated with laparotomy [10]. Endovascular therapy for AAO is an alternative treatment modality that is growing in popularity and showing promising results. In the study by Grip et al, thrombolytic therapy in combination with percutaneous transluminal angioplasty and/or stenting was used in nearly 25% of all AAO cases [9]. Endovascular therapy is the preferred treatment in patients with hypercoagulable states, like COVID-19, because this group of patients traditionally respond poorly to surgical intervention [9,11]. A popular endovascular therapeutic approach involves the use of the EkoSonic Endovascular System (EKOS) which emits targeted ultrasound waves that accelerates the dissolution of the fibrin matrix, and works in conjunction with tissue plasminogen activator (TPA) to achieve fibrinolysis.

Reperfusion injury results from the reintroduction of oxygen into a previously ischemic environment. This leads to the activation of endothelial cells and generation of free radicals by leukocytes, which together damages tissue. The inflammatory response causes a hypercoagulable state and vascular permeability. Electrolyte imbalances include hyperkalemia and acidosis. This leads to multisystem organ damage and cardiac arrhythmias [12]. Although there is research demonstrating promising results, at this time there is no clinically relevant treatment for reperfusion injury. Decreasing time to reperfusion, as well as monitoring fluid and electrolyte balances are key treatment strategies for decreasing mortality in reperfusion injury.

## Conclusions

COVID-19 can cause thrombus formation in unusual locations like the abdominal aorta. The in-situ thrombi can propagate to the point of causing complete vessel occlusion. EKOS thrombolysis serves as an effective alternative treatment to restore blood flow to previously ischemic tissue in patients who are poor surgical candidates. However, clinicians must be conscientious of reperfusion injury and potential sequelae.
